# The Moderating Effects of Perceived Control on the Relationship Between Childhood Trauma and Adult Cognitive Health

**DOI:** 10.1093/geroni/igaf122.3018

**Published:** 2025-12-31

**Authors:** Chloe Horowitz, Eric Cerino, Laura Noll, Nora Dunbar

**Affiliations:** Oregon State University, Corvallis, Oregon, United States; Northern Arizona University, Flagstaff, Arizona, United States; Northern Arizona University, Flagstaff, Arizona, United States; Northern Arizona University, Flagstaff, Arizona, United States

## Abstract

Trauma exposure has been shown to predict change in both executive function (EF) and episodic memory (EM), such that individuals with greater numbers of trauma exposure experienced greater rates of cognitive decline in both cognitive domains than those with fewer exposures. While it is clear that trauma exposure is a risk factor for cognitive decline in adulthood, it is unclear what factors may act as a buffer in this relationship and protect against the deleterious effects of childhood trauma. Perceived control is an important psychosocial resource for cognitive health that may serve as a buffer against these deleterious effects. This study seeks to examine the extent to which perceived control moderates the relationship between childhood trauma and cognitive health in adulthood. We hypothesized that the detrimental impact of childhood exposure to potentially traumatic events (PTE) on adult cognitive health will be attenuated among those with greater perceived control. We used data from waves 2 and 3 of the Midlife in the United States study. Results from multilevel models revealed that perceived control buffered the detrimental impacts of childhood PTE on cognitive health, but the buffering effect was specific to level and change in EF. Exploratory analyses revealed that higher levels of emotional neglect were associated with worse EF performance only among those with lower perceived control. Perceived control did not moderate effects of childhood PTE on EM outcomes. These findings characterize relationships between childhood PTE and cognitive health later in life by identifying the protective effects of perceived control.

